# Individuals with Autism Spectrum Disorders Do Not Use Social Stereotypes in Irony Comprehension

**DOI:** 10.1371/journal.pone.0095568

**Published:** 2014-04-18

**Authors:** Tiziana Zalla, Frederique Amsellem, Pauline Chaste, Francesca Ervas, Marion Leboyer, Maud Champagne-Lavau

**Affiliations:** 1 Institut Jean Nicod, CNRS, UMR 8129, Institut d'Etude de la Cognition, Ecole Normale Supérieure, Paris, France; 2 INSERM U 955, IMRB & University Paris Est Creteil, AP-HP, Henri Mondor-Albert Chenevier Hospitals, Department of Psychiatry, Fondation FondaMental, French National Science Foundation, Creteil, France; 3 Aix-Marseille University, CNRS, LPL UMR 7309, 13100 Aix-en-Provence, France; UCLA, United States of America

## Abstract

Social and communication impairments are part of the essential diagnostic criteria used to define Autism Spectrum Disorders (ASDs). Difficulties in appreciating non-literal speech, such as irony in ASDs have been explained as due to impairments in social understanding and in recognizing the speaker’s communicative intention. It has been shown that social-interactional factors, such as a listener’s beliefs about the speaker’s attitudinal propensities (e.g., a tendency to use sarcasm, to be mocking, less sincere and more prone to criticism), as conveyed by an occupational stereotype, do influence a listener’s interpretation of potentially ironic remarks. We investigate the effect of occupational stereotype on irony detection in adults with High Functioning Autism or Asperger Syndrome (HFA/AS) and a comparison group of typically developed adults. We used a series of verbally presented stories containing ironic or literal utterances produced by a speaker having either a “sarcastic” or a “non-sarcastic” occupation. Although individuals with HFA/AS were able to recognize ironic intent and occupational stereotypes when the latter are made salient, stereotype information enhanced irony detection and modulated its social meaning (i.e., mockery and politeness) only in comparison participants. We concluded that when stereotype knowledge is not made salient, it does not automatically affect pragmatic communicative processes in individuals with HFA/AS.

## Introduction

Social and communication impairments are part of the essential diagnostic criteria used to define Autism Spectrum Disorders (ASDs) [1], [2]. These impairments are often related to a serious deficit in the capacity for mentalizing [3], the natural tendency to explain everyday actions in terms of mental states. High Functioning Autism (HFA) and Asperger Syndrome (AS) are widely acknowledged to be variants on this spectrum [1], [2]. While HFA commonly refers to individuals with a history of speech and language delay, individuals with AS show no evidence of delayed language function. Moreover, differently from individuals with low-functioning autism, adults with HFA and AS (HFA/AS) do pass first- and second-order Theory-of-Mind or mindreading (i.e., the ability to attribute mental states to oneself and to others; ToM) tests [4]–[5], but they may fail in more ‘advanced’ ToM tasks, based on the detection of sarcasm, irony or bluff [6] or the recognition of Faux Pas [7], [8].

Using a computer-mediated communication program, Rajendran, Mitchell and Rickards [9] examined non-literal language comprehension in children and adolescents with HFA/AS and found that they were able to make appropriate responses in a verbal irony comprehension task. More recently, using a computer-mediated communication procedure, Glenwright and Agbayewa [10] confirmed that when verbal and social demands are minimized, children with HFA/AS are able to perform as well as control participants on measures of verbal irony comprehension, such as judging the speaker's intentions and irony’s social function of conveying humour.

However, Jolliffe and Baron-Cohen [11] reported that although adults with HFA are able to grasp the speaker’s ironic intent (i.e., that the speaker meant something other than the literal meaning), they may encounter difficulties processing mental state information and are less able than control subjects to use contextual information to justify the speaker’s utterance. Along the same lines, in a neuroimaging study, Wang and collaborators [12] found good accuracy at irony detection along with a different pattern of neural activity in children with HFA, as compared to the typically developed group, when trying to incorporate contextual information in order to make inferences about speaker ironic intent. Specifically, the authors interpreted the significantly greater activity observed in the right inferior frontal gyrus and in the temporal regions bilaterally as reflecting different strategies for irony comprehension and greater cognitive demands in children with HFA.

It has been shown that irony comprehension requires the acquisition of the shared knowledge and communicative strategies adopted by a social community [13]. Irony is regarded as a more polite, and indirect, way of expressing a speaker’s criticisms [14]–[16]. In the case of an ironic insult, the most widespread form of sarcasm, the speaker’s attitude is regarded as being more *positive* than it is for its literal counterpart [17]. Such positiveness, known as the “Tinge Effect” [17], [18] would be induced by the obligatory processing of the literal (positive) interpretation of ironic statements in negative situations, which would attenuate the negativity expressed by these statements, when ironically interpreted.

Various sources of information might influence the modulation of the overall meaning of the ironic comment. Irony understanding depends in part on the strength of the social cues contextually available for inferring the speaker’s intent. According to Colston [19], the degree of negativity expressed by an ironic comment would depend on how critical the situational context perceived by the listener is, relative to the positiveness of the comment, ironically intended by the speaker. Hence, as proposed by Fussell and Kreuz [20], both psycholinguistic processes (e.g., lexical retrieval, syntactic processing) and social-interactional factors affect irony interpretation. Stereotypes are energy-saving devices associated with an automatic, unintentional, and unconscious process [21]; they guide expectations, inferences, and impressions, and shape interpretations and memory retrieval [22].

Remarkably, Pexman and Olineck [23] showed that speaker characteristics conveyed by an occupation stereotype are integrated in the linguistic comprehension process and may cue ironic intent when other contextual cues are minimal. For example, the effect of occupation stereotype information on irony comprehension has been explained by what people imagined about target individuals [24]. Specifically, an occupation stereotype affects irony comprehension if that occupation is strongly consistent with speaker behaviour. Similarly, it has been shown that people recall information better and more readily when it is consistent with a preexisting stereotype than when it is inconsistent [25]. Speakers’ propensities to be humorous, mocking, less sincere and more prone to criticism act as salient cues for ironic intent. Thus, such speaker characteristics facilitate irony detection by indicating that the speaker is likely to have a negative attitude and that such an attitude would be indirectly expressed through humour and insincerity. Conversely, if the speaker’s occupational stereotype is consistent with sincerity and seriousness, the listener might be less likely to detect the speaker’s ironic intent [26]–[28]. All these features contribute to setting up a specific “ironic context” and enhance the speaker’s ironic attitude [29].

A speaker’s social stereotype (e.g., occupation, gender and ethnic group) also influences memory for and interpretation of potentially ironic remarks at a very early stage in the process of sarcasm and irony comprehension [23], [30]. Using online reading tasks, Pexman, Ferretti and Katz [28] reported that when speaker occupation was mentioned, reading times were longer at the statement’s end indicating that speaker occupation knowledge is integrated with lexical and syntactic information relatively early, even in the absence of an explicit decision about speaker intent. By using a series of puppet show scenarios, Pexman and collaborators [13] reported that children with HFA exhibited spared competence for irony comprehension, though group differences in processing strategies, in terms of gaze behavior and response latencies, likely reflected a less elaborate understanding of others' communicative intents. Notably, they failed to detect the intended humor conveyed by ironic criticisms and failed to appreciate the broader social function of irony, such as the speaker’s intent to be simultaneously critical and humorous.

Recently, Hirschfeld and collaborators [31] have showed that ToM abilities are not crucial for acquiring social stereotypes, but they might be used to overcome them. Individuals with ASDs who have diminished mindreading capacities might find it easier to understand others as group members using stable character traits rather than explaining human behaviour by processing the large variety of detailed information available during on-line social interaction. Indeed, according to White and collaborators [32], the propensity to make judgments based on race and gender stereotypes in adults with AS is similar to that for typically developed populations, suggesting that the use and acquisition of stereotype knowledge can proceed along with diminished social engagement and ToM abilities. However, while this study provides evidence about the ability to use stereotypic knowledge in explicit tasks, it does not tell us to what extent this knowledge is spontaneously used in everyday communication.

Rumsey and Hamburger [33] proposed that a core deficit of autism consists in a broad class of verbal and nonverbal conceptual reasoning disabilities. In the same line, Minshew and Goldstein [34] put forward a multiple primary cognitive deficit model that described the cognitive profile of individuals with autism spectrum disorder as a disorder of complex information processing across cognitive domains. Complexity is defined in terms of the number of elements contained in the stimulus material as well as the multiplicity of cognitive processes involved in task performance. Within a cognitive theory, complex information processing requires the integration of multiple features and the reliance on different component processes. Across domains, complex information processing theory provided an explanation for the particular constellation of deficits that define ASDs, including impairments in concept formation, complex memory, complex language, and skilled motor abilities. The consequences of impaired complex information processing in ASDs would be also manifested in real-world situations, as this population will experience difficulty in fast dynamic social interactions because of their inability to quickly process relevant information on line.

In the present study, we aimed to investigate whether, as has previously been shown for typically developed subjects [23], social stereotype information can be used to enhance irony detection in individuals with HFA and AS. Given their difficulties in using context information and integrating them during communication and on-line comprehension processing one might expect that stereotype information would not enhance the propensity in individuals with ASDs to interpret an utterance as ironic in those circumstances in which the stereotype would favour an ironic interpretation (i.e. a sarcastic occupation). To investigate whether individuals with HFA or AS can use and integrate occupational stereotype knowledge in an irony comprehension task, we used a series of verbally presented stories containing either a sarcastic-ironic or literal statement uttered by a speaker characterised as having a “sarcastic” (i.e., perceived as more prone to use sarcasm) or “non-sarcastic” occupation. As already reported [26], people perceive speakers with certain occupations as being more likely to use irony than speakers with other occupations.

Given that the ability to process and integrate different types of information on-line rapidly and efficiently might be impaired in this population, one could also expect a reduced effect of stereotype knowledge on pragmatic processes underlying irony comprehension. Verbal irony can serve many social functions: Speakers can temper the aggression conveyed by criticism, or praise conveyed by a compliment (the Tinge Hypothesis) [35], while bringing humour to a situation. A full understanding of ironic language requires one to make complex inferences about speaker intent, a task that can be challenging for individuals with ASDs who might have difficulties with mentalizing in social contexts or with the interpretation of ambiguous stimuli. According to Pexman and Olineck [18], the degree of perceived irony would be a way to assess listeners’ ToM abilities while the degree of perceived politeness would be a way to assess their social competence more broadly. In the present study, we also assessed whether occupational stereotype would influence recognition and apprehension of the communicative (i.e., mocking) and social features of irony (i.e., politeness). It is possible that they would fail to fully appreciate the social function of irony, such as its mocking and positive dimensions, indicating difficulties with pragmatic processing. Such subtle differences in pragmatic understanding may underlie some of the social difficulties faced by individuals with ASDs.

## Materials and Methods

### Participants

Seventeen adults with a clinical diagnosis of High Functioning Autism (HFA) (N = 6) or Asperger Syndrome (AS) (N = 11) according to DSM-IV R (American Psychiatric Association, 2000) and ASDI (Asperger Syndrome Diagnostic Interview) [36], were recruited from Albert Chenevier Hospital in Créteil (Table 1). The inclusion and exclusion criteria for the clinical group were based on retrospective parental information about the early language development of their child. All diagnoses were made by experienced clinicians and were based on clinical observations of the participants. Interviews with parents or caregivers using the ADOS (The autism diagnostic observation schedule-generic) [37] and the ADI-R (Autism Diagnostic Interview) [38] confirmed the diagnoses. The cut-off points for the three classes of behaviour for the ADI are a score of 10 for reciprocal social interaction [B], 8 for communication [C], and 3 for stereotyped behaviours [D], respectively. All participants scored above the cut-off points.

**Table 1:** Means (and standard deviations) of demographic and clinical data for participants with HFA/AS and the comparison participants.

	HFA/AS	Comparison
**N (male:female ratio)**	14∶3	12∶5
**Age in years (mean, SD, range)**	27.3 (7.3); 18–40	30.1 (9.7); 20–47
**Education in years (mean, SD)**	13.4 (3.8); 8–18	13.5 (2); 10–18
**ADI [B,C,D]** *	18.6 (6.8); 11.6 (6.6); 6.9 (3.2)	−
**Full-scale IQ**	93.7 (21.1); 70–137	96.2 (10.9); 80–116
**Verbal IQ**	99.3 (20.1); 70–143	98.7 (9.9); 85–123
**Performance IQ**	90.7 (18.4); 70–122	95.9 (10.3); 80–118

* [B] = reciprocal social interaction, [C] = communication, [D] = stereotyped behaviours.

Seventeen typically developed comparison participants (CP) volunteered to match the clinical group with respect to age, IQ and gender (Table 1). Prior to their recruitment, the comparison participants were screened to exclude any with a history of psychiatric or neurological disorders. All participants were native French speakers, and had normal/corrected to normal vision. All participants received basic neuropsychological screening, which included Verbal and Performance IQs (WAIS-III) [39]. All participants had an IQ above 70. Overall, individuals with HFA/AS did not differ from the comparison participants on gender, chronological age (t-test: t(32) = 1.14, *p = *0.26), education (t-test: t(32) = 0.17, *p = *0.87), Full-scale (t(32) = 0.43, *p* = 0.7), verbal (t(32) = −0.12, *p* = 0.90) and Performance (t(32) = 1.1, *p* = 0.31) (Table 1).

The present research has been approved by the local Ethical committee (Inserm, Institut Thématique Santé Publique; C07-33). All participants signed informed consent agreements before volunteering for this study, and all investigation complied with APA ethical standards.

### Procedure

Before running the main experiment, a pilot study was conducted on a preliminary set of 45 occupations. Forty French native speakers were required to rate the probability that a person with a given occupation would make an ironic utterance using a 1 (low probability) to 7 (high probability) rating scale. All of these participants were chosen from the general population (mean age: 29, SD 4.2) and did not participate in the experiment. The following seven occupations were judged to have the highest probability of ironic remarks (sarcastic occupations): *comedian*, *talk show host*, *actress*, *artist*, *mechanic*, *plumber* and *insurance agent*. The following seven occupations were judged to have the lowest probability of ironic remarks (“non-sarcastic occupations”): *accountant*, *clergyman*, *scientist*, *librarian*, *waiter*, *bank teller* and *veterinarian.*

Participants in the experiment were individually tested in a quiet room at the Albert Chenevier Hospital in Créteil. In line with Pexman and Olineck’ study [23], twenty-one pairs of stories, containing either an ironic or a literal utterance were visually presented on a computer screen with no time limit. Each statement (ironic or literal) was uttered by a speaker having either a sarcastic occupation or a non-sarcastic occupation within seven stories (see Table 2 for examples). A no occupation condition, in which the speakers were only identified by surname, was included to rule out the possibility that participants would adopt a response strategy of predicting job information for each story. Testing time varied from approximately 45 to 60 minutes. The test consisted of two separate sessions with a short interval in between (15 to 20 min). Additional breaks or refreshments were given when requested. Stories having the same context and the same speaker uttering an ironic or a literal statement were presented in different sessions. Within each session, stories were presented in randomized order to avoid order effect, and session presentation was counterbalanced across subjects. Stories were visually presented on a computer screen and were available throughout the experiment. Participants were asked to read each story attentively before answering questions. There was no time limit.

**Table 2:** Examples of story types.

Stories	Context	Literalstatement	Ironicstatement
1. Stories with speakershaving a sarcasticoccupation	*Marie-Eve told his friend, an actor (sarcastic occupation), that she could* *memorize a poem of 20 lines in 5 minutes. Marie-Eve recited only half a* *poem and forgot the rest.* ***The day after, the actor says to Guillaume:***	*Marie-Ève has a* *mediocre memory.*	*Marie-Ève has a* *phenomenal memory.*
2. Stories with speakershaving a non-sarcasticoccupation	*A veterinarian (non-sarcastic job) sees Joannie arriving at work on* *Monday morning. Joannie seems to be a little bit more tired than usual.* ***At midday, the actor says to Pierre:***	*Joannie does not* *look well.*	*Joannie looks well.*
3. Control stories withspeakers with nooccupation	*Louise (no job) has moved house today and Michel has told her that* *he will come to help her all day. Michel comes to help Louise* *but just for a few minutes.* ***The following day, Louise says to Amélie:***	*Michel is* *uncooperative.*	*Michel is helpful.*

The experimenter introduced the experiment as follows: ‘Here, on this computer screen, you will be presented with a series of stories describing social situations with one person, the speaker, expressing a judgment about another person. You are to read each story presented on this computer screen for as long as you wish. When you are finished reading the whole story, you will be asked to answer some questions. If you think that everything is clear and feel ready to start, you should press the space bar on the computer keyboard”. Participants were invited to ask clarification questions; if needed, the experimenter could also read the stories. The experimenter sat close to the subject. The computer screen was placed in front of the participant and each story remained available throughout the reading and questioning. A training session of five trials preceded the experiment, and allowed participants to familiarise themselves with the task and with the response measures. Specifically, participants were required to use the rating scales and ask for clarifications. The following definition of irony was given: *Verbal irony is a statement in which what the speaker means is different from what he/she ostensibly states*. The experimenter also provided a few examples of verbal irony (e.g. “The speaker says “It’s a lovely day,” in a downpour of rain). Consistency between responses to the first question (“Speaker Intent Question”) and irony rating was also used to ensure that participants fully understood how to use the scales and the notion of “irony”.

The first question, the “Speaker Intent Question” (“*What does the speaker actually mean?*”) allowed us to assessed accuracy at irony detection, that is the ability to understand whether the last remark in the story had been intended as ironic or literal, by a forced choice question (“*Does he mean that Marie-Ève has a good memory?*” *or* “*Does he mean that Marie-Ève does not have a good memory?*”).

Then, using four 7-point rating scales, participants had to judge whether the speaker was *ironic* (ranging from 1 =  not at all ironic to 7 =  extremely ironic), *mocking* (1 =  not at all mocking and 7 =  extremely mocking), and *polite* (1 =  not at all polite and 7 =  extremely polite). The last question, a control question, was specific to each story and allowed us to verify that participants had not gotten confused or forgotten important details of the story.

The story was placed in front of the participant and remained there throughout the reading and questioning so that participants did not have to remember it. This was done in order to minimize memory and attention requirements.

### Data Collection and Analyses

Conformity to the assumptions of parametric statistics was assessed using the Komolgorov-Smirnov Normality test (χ^2^(2, N = 39) = 1.42, p = 0.98), to check that the data came from normally distributed samples and the F-test was used for equality of variances (F(21,16) = 0.49, p = 0.14).

The data were analysed using repeated-measures ANOVAs with factors Groups (2: CPs, HFA/AS) X Statement (2: ironic, literal) X Speaker’s occupation (2: non-sarcastic, sarcastic). Scheffe’s tests were used for post-hoc analysis. Pearson product-moment correlation coefficients were calculated across all participants between clinical measures and test results. Measures of effect size were calculated for each effect of interest by providing the Partial Eta-squared for ANOVAs and Cohen's **d** for t-test. The level of significance was at <0.05.

## Results

### Speaker Intent

A forced choice question assessed whether participants correctly recognized that speakers made literal or ironic compliments within a given context. Overall, 80% of CP and 72.5% of HFA/AS correctly recognized the speaker’s communicative intent, that is whether the utterance was to be interpreted as ironic or literal.

**Figure 1:**
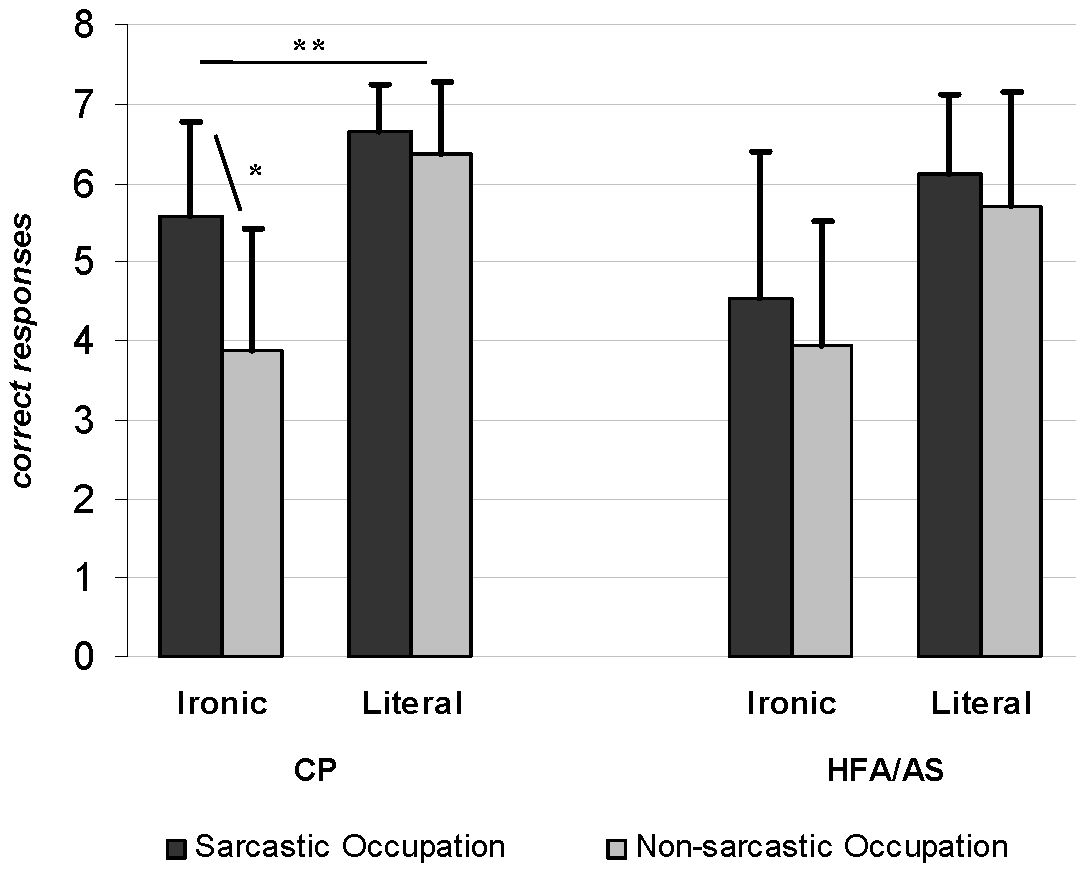
Number of correct responses produced by the two participant groups as a function of the statement (Ironic and Literal) and of the speaker (Sarcastic and Non-sarcastic). Error bars are standard deviations. * p<.0005; ** p<.0001.

Repeated-measures ANOVA yielded a marginally significant main effect of Group (F(1,32) = 2.9; p = 0.09, η_p_^2^ = 0.09) and significant main effects of Statement (F(1,32) = 34.4; p<0.0001, η_p_^2^ = 0.51) and Occupation (F(1,32) = 22.6; *p*<0.0001, η_p_^2^ = 0.41). The effect of Statement was due to both groups performing significantly better in the literal condition than in the ironic one (mean difference = −1.7; *p*<0.0001), while the Occupation effect was due to the greater number of correct responses on the speakers’ sarcastic occupation condition, as compared to the speaker’s non-sarcastic occupation condition (mean difference = −0.75; *p*<0.0001). These effects were qualified by a significant Statement X Occupation interaction (*F*(1,32) = 10.9; *p = *0.002, η_p_^2^ = 0.25), and a significant Statement X Occupation X Group interaction (*F*(3,96) = 6.6; *p = *0.01, η_p_^2^ = 0.17). The significant Statement X Occupation interaction was due to the greater number of correct responses for the sarcastic occupation as compared to the non-sarcastic occupation in the ironic statement condition (mean difference = −1.14; *p* = 0.006), while this difference was not significant in the literal condition (mean difference = −0.35; *p* = 0.17). The Statement X Occupation X Group interaction further revealed that this difference was significant only for the CPs (*p* = 0.001) and not for the group with HFA/AS (*p* = 0.37) (Figure 1). The Occupation X Group (*F*(1,32) = 2.5; *p* = 0.12, η_p_^2^ = 0.07) and the Statement X Group (*F*(1,32) = 0.02; *p* = 0.88, η_p_^2^ = 0.0007) interactions were not significant.

### Irony Rating

Irony ratings were analyzed only for those trials on which participants correctly responded to the speaker’s intended meaning. For ratings of the extent to which a speaker was being ironic, there were highly significant main effects of Statement (*F*(1,32) = 87.7; *p*<0.0001, η_p_^2^ = 0.72) and Occupation (*F*(1,32) = 13.5; *p* = 0.0009, η_p_^2^ = 0.30), as well as a significant Statement X Occupation interaction (*F*(1,32) = 8.2; *p* = 0.007, η_p_^2^ = 0.20), a group X occupation interaction (*F*(2,64) = 4.2; *p* = 0.047, η_p_^2^ = 0.11) and a significant Statement X Occupation X Group interaction (*F*(3,96) = 5.0; *p* = 0.032, η_p_^2^ = 0.13). The main effect of Group (*F*(1,32) = 0.3; *p* = 0.56, η_p_^2^ = ) and the Statement X Group interaction (*F*(1,32) = 1.6; *p* = 0.21, η_p_^2^ = 0.04) were not significant.

**Figure 2:**
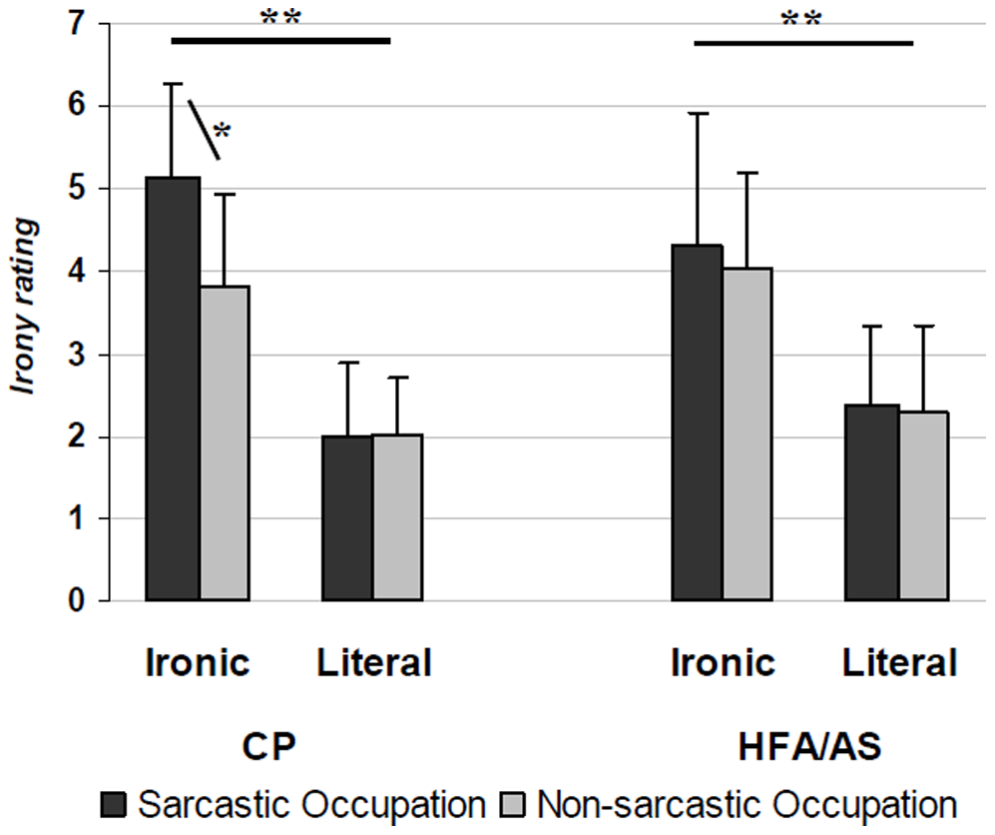
Irony ratings (1 =  not at all ironic and 7 =  extremely ironic) of the speaker statement by the two participant groups as a function of the statement (Ironic and Literal) and of the occupation (Sarcastic and Non-sarcastic). Error bars are standard deviations. * = p<.05; ** = p<.0001.

For both groups, irony ratings were higher when the statements were ironic as compared to literal (mean difference = 2.1; *p*<0.0001), while the Occupation effect was due to the higher irony ratings when the speakers had a sarcastic occupation as compared to speakers with non-sarcastic occupations (mean difference = −0.42; *p* = 0.0009). As revealed by the significant Statement X Occupation interaction, this difference was only significant in the ironic statement condition (*p* = 0.01) and not in the literal one (*p* = 0. 89). Moreover, the Statement X Occupation X Group interaction effect revealed that, in the irony statement condition, the irony ratings were higher for speakers with sarcastic occupations than for speakers with non-sarcastic occupations only for the CPs (*p* = 0.001), while the difference was not significant for the HFA/ASs (*p* = 0.54). Mean rating of ironic utterances pronounced by sarcastic speakers was higher for CPs than for participants with HFA/AS (*p* = 0.05) (Figure 2).

### Mockery Rating

Mockery ratings were analyzed only for those trials on which participants correctly responded to the speaker’s intended meaning. Repeated-measures ANOVA yielded a main effect of Statement (*F*(1,32) = 36.2; *p*<0.0001, η_p_^2^ = 0.55) and Occupation (*F*(1,32) = 14.5; *p* = 0.0006, η_p_^2^ = 0.34), as well as significant interaction effects of Statement X Occupation (*F*(1,1) = 8.7; *p* = 0.006, η_p_^2^ = 0.17) and Occupation X Group (*F*(2,64) = 5; *p* = 0.03, η_p_^2^ = 0.12). The group difference was not significant (*F*(1,32) = 1.5; *p* = 0.22, η_p_^2^ = 0.07), nor were the Statement X Group (*F*(1,32) = 2.6; *p* = 0.12, η_p_^2^ = 0.07), and Statement X Occupation X Group (*F*(3,96) = 1.3; *p* = 0.27, η_p_^2^ = 0.03) interactions significant.

**Figure 3:**
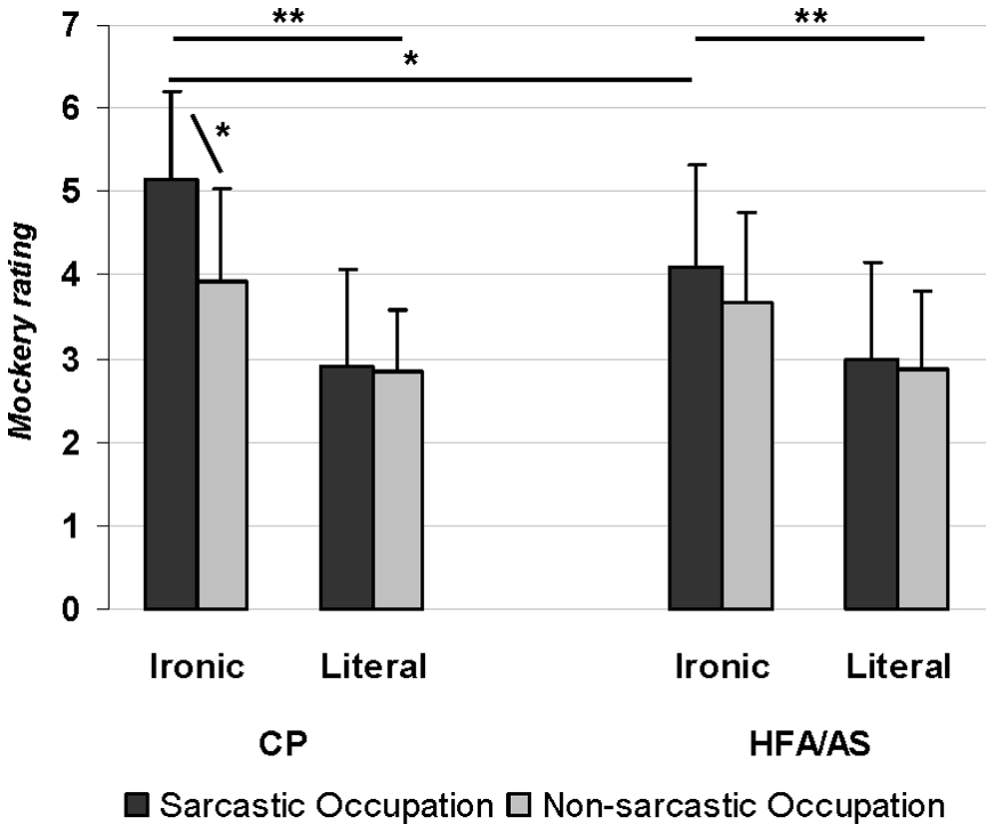
Mockery ratings (1 =  not at all mocking and 7 =  extremely mocking) of the speaker statement by the two participant groups as a function of the statement (Ironic and Literal) and of the occupation (Sarcastic and Non-sarcastic). Error bars are standard deviations. * = p<.05; ** = p<.0001.

Ironic statements were rated as more mocking than the literal statements (mean difference = 1.3; *p*<0.0001) and the statements uttered by the speakers with a sarcastic occupation were rated as more mocking than the statements uttered by speakers with a non-sarcastic occupation (mean difference = −0.41; *p* = 0.0006), However, as revealed by the Statement X Occupation and the Occupation X Group interactions, the difference between the mocking ratings produced by the two types of speakers was significant only for the ironic statement condition (*p* = 0.004), and for the comparison group (*p* = 0.02), but not for the HFA/AS group (*p* = 0.28). Mean rating of mockery for utterances pronounced by sarcastic speakers was significantly higher for CPs than for participants with HFA/AS (*p* = 0.01) (Figure 3).

### Politeness Rating

Politeness ratings were analyzed only for those trials on which participants correctly responded to the speaker’s intended meaning. Repeated-measures ANOVA yielded significant main effects of Statement (*F*(1,32) = 70.9; *p*<0.0001, η_p_^2^ = 0.56) and Occupation (*F*(1,32) = 25.7; *p*<0.0001, η_p_^2^ = 0.34), as well as a significant Occupation X Group interaction (*F*(2,64) = 18.1; *p* = 0.0002, η_p_^2^ = 0.34) and a Statement X Occupation X Group interaction (*F*(3,96) = 4.5; *p* = 0.04, η_p_^2^ = 0.17). No significant main effect of Group (*F*(1,32) = 0.4; *p* = 0.50, η_p_^2^ = 0.02) was found. Neither Statement X Occupation (*F*(1,32) = 0.6; *p* = 0.4, η^2^ = 0.02) nor Statement X Group (*F*(2,64) = 0.001; *p* = 0.97, η_p_^2^ = 0.0007) interactions were significant.

**Figure 4:**
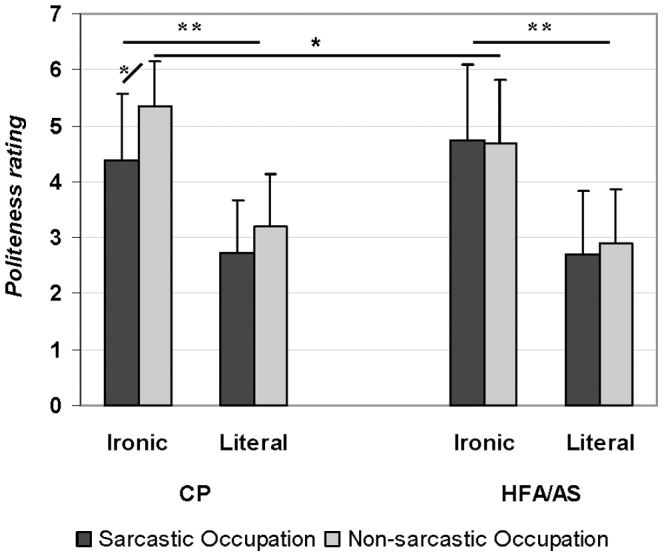
Politeness ratings (1 =  not at all polite and 7 =  extremely polite) of the speaker statement by the two participant groups as a function of the statement (Ironic and Literal) and of the occupation (Sarcastic and Non-sarcastic). Error bars are standard deviations. * = p<.05; ** = p<.0001.

The effect of Statement was due to the ironic statements being rated as more polite than the literal statements (mean difference = 1.9; *p*<0.0001), while the Occupation effect was due to the statements uttered by the speakers with non-sarcastic occupation being rated as more polite than the ones uttered by the speakers with sarcastic occupation (mean difference = 0.3; *p*<0.0001). Post-hoc Scheffe’s tests revealed that this difference was significant only for the CPs (*p* = 0.02), while participants with HFA/AS rated ironic statements pronounced by the two types of speaker as equally polite (*p* = 0.86). Mean rating of politeness for ironic utterances pronounced by non-sarcastic speakers was significantly higher for CPs than for participants with HFA/AS (*p* = 0.05) while there was no significant group difference in the other conditions (Figure 4).

### Control Questions

No group difference was found on the number of correct responses for the control questions on the two pairs of twenty-one stories (*t*(1,30) = 0.7, *p = *0.47; Cohen's *d* = −0.30). Both participants with HFA/AS (mean = 41.5±0.8; range = 40–42) and comparison participants (mean = 41.7±0.5; range = 41–42) responded equally to the control questions. Overall, participants correctly understood the stories. The few errors committed concerned irrelevant details that did not crucially affect task performance (e.g., “*Samuel arrived two hours late at the meeting”* instead of “*Samuel arrived one hour late at the meeting”* or *“The first course was too salty” instead of “The main course was too salty”*).

### Occupation Stereotype Judgment

To assess whether, like the comparison group, participants with HFA/AS possess acquired occupation stereotype knowledge or whether they are unable to use it in the context of irony understanding, in a subsequent session we asked participants with HFA/AS to rate the probability that persons with the occupations used in the experimental task would make a sarcastic utterance, a criticism, a mocking, or a polite remark by using a 1 (low probability) to 7 (high probability) rating scale. The sarcastic occupations (i.e., *comedian*, *talk show host*, *actress*, *artist*, *mechanic*, *plumber* and *insurance agent)* were judged as having a higher probability of sarcasm (mean rating = 4.2, ±0.2; t(16) = 7.7; p<0.0001; Cohen's *d* = 3.1), criticism (mean rating = 4.4, ±0.8; t(13) = 5.7; p<0.0001; Cohen's *d* = 1.8), mocking (mean rating = 4.4, ±0.9; t(13) = 8.4; p<0.0001; Cohen's *d* = 1.5) and less polite (mean rating = 5.3±0.8; t(13) = −4.6; p = 0.0004; Cohen's *d* = −1.2) remarks than the non-sarcastic occupations (i.e., *accountant*, *clergyman*, *scientist*, *librarian*, *waiter*, *bank teller* and *veterinarian)* (Mean rating for sarcastic = 2.4, ±0.8; criticisms = 2.87, ±0.9; mocking = 2.6, ±1.4 and less polite = 6.1±0.5) remarks. The evaluation of each occupational category for irony propensity did not differ from that provided by a group of 17 age and gender matched subjects participating to the pilot study for both the sarcastic (mean rating = 4.4±0.6; t(32) = 0.8; p = 0.41 Cohen's *d* = 0.4) and non-sarcastic ones (mean rating = 2.5±0.4; t(32) = 0.4; p = 0.7; Cohen's *d* = 0.1).

## Discussion

The present study aimed to investigate irony comprehension and understanding of social occupational roles in individuals with HFA/AS, and whether these two sources of knowledge would be processed in an integrated manner in communicative tasks. The results showed that participants with HFA/AS exhibited no difficulties understanding irony (i.e., utterances having a meaning that is the opposite of the literal meaning), confirming relatively preserved abilities to perform pragmatic reasoning tasks [10], [40], [41]. In fact, HFA/AS and comparison groups recognized a comparable number of ironic utterances and, accordingly, for both groups, the level of irony assigned to the ironic utterances was higher compared to the literal ones. Overall, participants performed significantly better in the literal condition than in the ironic one, confirming the claim that irony is more difficult to understand than literal language and makes greater cognitive demands [42], [43].

Verbal irony plays an important role not only in conveying attitudes, but also as a reminder of moral, social and aesthetic norms tacitly shared by a culturally-defined social group [14], [35]. Importantly, a full understanding of irony requires some appreciation of why the speaker has chosen this communicative strategy to express her thought. Irony is generally perceived as a less negative, more humorous and polite manner of expressing criticism than its literal paraphrases [23], [26], [27], [29], [35]. According to the echoic account [44], [45], the point of irony is not to commit the speaker to the truth of the proposition expressed by the statement, but rather to express a certain type of derisory or dissociative attitude to a tacitly attributed thought. In other words, the speaker in irony mode is *echoing* a thought she attributes to someone else, while she conveys her mocking, sceptical or contemptuous attitude to that thought. Processing such a *tacit* dissociative attitude requires metarepresentational and social abilities that, according to the present findings, appear to be preserved in our group of adults with HFA or AS.

In a recent study, Pexman and collaborators [13] showed unimpaired irony comprehension, but difficulties with the appreciation of the intended humor conveyed by ironic criticisms in children with HFA. These results are not in accordance with the present findings showing that adults with HFA were able to appreciate the social features of irony. However, the two experimental groups have different chronological ages and this might help explain the improvement in adults’ performance. Indeed, it is likely that greater practice with social situations would play an important role in increasing the understanding of social norms in individuals with HFA/AS.

The present results reveal that occupation stereotype information modulates and improves irony detection only in comparison participants. As previously shown [18], [28], the speaker’s sarcastic occupation (e.g., *actor*, *talk show host*), which is associated with psychological traits and propensities consistent with irony, enhances detection of ironic intent, whereas a non-sarcastic occupation (e.g., *clergyman*, *scientist*) - being inconsistent with an ironic interpretation of that utterance - did not favor such an interpretation in comparison participants. Consistently, speaker’s sarcastic occupation increased ratings of the speaker’s ironic and mocking attitude with typically-developed adults. In contrast, for individuals with HFA/AS, speaker’s occupation stereotype did not enhance accuracy performance in irony comprehension and did not modulate irony and mockery ratings as a function of the speakers’ occupation. Given these expectations elicited by occupation stereotypes, typically-developed adults also regarded an ironic criticism expressed by a speaker with a sarcastic occupation as being less polite than the same insults expressed by speakers with a non-sarcastic occupation, showing that knowledge about the stereotypical traits associated with the speakers’ occupation influenced other pragmatic and communicative processes related to the social function of irony. Such an effect seems to be absent in participants with HFA/AS who, despite their overall spared ability to understand irony and its social functions (i.e., irony is considered as being more mocking and polite than literal utterances), attributed equal level of mockery and politeness to both types of speakers. However, when explicitly asked to rate the probability that a person having one of the occupations used in the experimental task would make a sarcastic, a humoristic and a polite remark, participants with HFA/AS exhibited a propensity to perceive some occupations as being more ironic, sarcastic, mocking and polite than others, similarly to typically developed individuals. The present results point to a preserved ability to acquire and retrieve social occupational stereotypes in an explicit way, although such knowledge is not integrated in pragmatic reasoning in participants with HFA/AS to the same extent as in the comparison group.

Social stereotypes are cognitive structures (sets of associated beliefs) stored in long term semantic memory, containing large networks of abstract information about traits, attributes and expected behaviors of members of social groups. Stereotype information can be automatically activated in the presence of stimulus cues in the environment, such as a member of the stereotyped group or some symbolic equivalent. It does not require conscious effort when it exerts an influence on the encoding and interpretation of behaviour. This automatic stereotype processing involves unintentional or spontaneous activation of a well-learned set of associations or responses that have been acquired through repeated experience, while the controlled stereotype-related processes may exert a modulatory or inhibitory effect on automatically activated stereotypes [22]. Social stereotype knowledge might be part of a dedicated cognitive neural system, functionally dissociable from comparable classes of information in the brain, which stores and processes abstract person-based knowledge [46]. This dedicated knowledge memory system serve a fundamental aspect of social-cognitive functioning that might have evolved to deal with socially relevant information [47], since it allows making general prediction about people behaviours, when prior experience or on-line interaction are reduced.

Using a task of attribution of trustworthiness, attractiveness, socioeconomic status and age, White and collaborators [32] reported a preserved ability to make social stereotype judgments from photographs in participants with AS, despite their impairments in facial perception and mentalizing. Similarly, Hirschfeld and collaborators [31] showed intact reasoning about social groups in children with autism, since they performed like typically developing children in using race and gender stereotypes to predict behaviors in new contexts. Importantly, these studies focused on explicit measures to assess sensitivity to stereotypes in ASDs, such as asking the participants directly to make person judgments based on group membership.

Indeed, recent studies have shown that stereotype does not affect behaviour and attitudes only when one is required to make an explicit judgment about the speaker. There is substantial evidence that such information is automatically activated in the presence of a member or symbolic equivalent of the target group [22] and that the use of in social communicative tasks mostly relies on automatic and involuntary processes. As previously revealed [18], when participants were not asked to make an explicit decision about speaker intent, reading time measures showed that speaker occupation information is integrated at an early stage of statement processing. The same was likely the case in the present experiment in which the speaker’s occupation is never overtly elicited during task completion.

Thus, the present findings show spared abilities to form and retrieve social stereotype knowledge, along with a reduced automatic effect of stereotype information upon pragmatic-inferential reasoning in participants with HFA/AS. It is possible that failure to integrate information from distinct special-purpose mechanisms, such as the “Naïve Sociology” system, implicated in processing knowledge about social groups, and the “Naïve Psychology” system responsible for mind-reading [39] might lead to longer response times in computing on-line pragmatic-inferential processes during social interaction, in people with ASDs. This explanation is consistent with the Minshew and Goldstein [34]'s cognitive model which regards ASDs as a complex information processing disorder affecting higher-order inferential cognitive ability.

It is noteworthy that depending on circumstances, as stereotype attitudes are simplified conceptions of persons and groups, they can also be maladaptive and originate prejudices. In accordance with our findings, a recent study using the Implicit Association Test [48], a computerized classification paradigm, showed that participants with ASDs are less affected by the influence of stereotypes than typically developed controls [49]. Therefore, since individuals with ASDs are less prone to use automatic stereotypes, one might expect a reduced conformity to social norms and reduced stereotype attitudes in these individuals, relative to typically developed individuals.

## Conclusions

The present study confirms that stereotype knowledge is spontaneously activated during the psycholinguistic processing involved in irony comprehension and that information about ironic intent is combined with pragmatic and social information in typically developed subjects. In particular, stereotype knowledge exerts its influence on subjects’ expectancies by enhancing irony detection and its social traits in an implicit manner. In contrast, although adults with HFA/AS possess a preserved ability to understand irony and exhibit a developed capacity for understanding social stereotypes, when this knowledge is not made salient, it is not integrated and used in pragmatic communicative processes. Therefore, while we conclude that irony comprehension is preserved in adults with HFA/AS, occupational stereotypes do not appear to engage those automatic processes that, in some circumstances, might enhance detection of the speaker’s communicative intents and attitudinal features. Such reduced automaticity exerted by stereotype information might explain some of the impairments in rapid communication and on-line social interaction in individuals with HFA or AS. Hence, it is possible that the use of this information in social and communicative reasoning would occur only when it is previously activated through explicit and controlled processes.

Further studies are needed to investigate how different types of stereotypes are encoded and activated, as well as how this information covertly and overtly interacts with social reasoning and perception in individuals with ASDs.
